# Supplementary material for: Assessing the public health impact of tolerance-based therapies with mathematical models
Source: PLoS Comput Biol. 2018 May 4;14(5):e1006119. doi: 10.1371/journal.pcbi.1006119 (PMC5955582; doi:10.1371/journal.pcbi.1006119)
Supplement: S1 Text — (PDF) [file pcbi.1006119.s001.pdf]

Supporting Information: Assessing the public health impact of tolerance-based therapies with mathematical models

N. Hoze, S. Bonhoeffer, R. Regoes

Text A: Equilibrium analysis of the tolerance-based treatment model

In the numerical applications presented in Figs 1 and 2, we chose the transmission coefficient to be the same with and without treatment. Here, we consider almost the same model as (1), where we add for the sake of generality a cost of transmission c_T associated to treatment. The system of equations is now

$$\begin{aligned}\dot{S} &= \Lambda - \delta S - \beta S(I + (1 - c_T)T) \\ \dot{I} &= \beta S(I + (1 - c_T)T) - (\delta + v)I - \theta I - rI \\ \dot{T} &= \theta I - (\delta + v_T)T - r_T T \\ \dot{R} &= rI + r_T T - \delta R.\end{aligned} \tag{1}$$

The system of equations (1) has two equilibria, a disease-free equilibrium (DFE) given by $(S, I, T, R) = (\frac{\Lambda}{\delta}, 0, 0, 0)$ and the endemic equilibrium (EE)

$$\begin{aligned}
S^{eq}(EE) &= \frac{A_T\,(A+\theta)}{\beta\,(A_T + (1-c_T)\theta)} \\
I^{eq}(EE) &= \delta\frac{S^{eq}(DFE) - S^{eq}(EE)}{A+\theta} \\
T^{eq}(EE) &= \frac{\theta}{A_T} I^{eq} \\
R^{eq}(EE) &= \frac{1}{\delta}\left(r + r_T\frac{\theta}{A_T}\right) I^{eq}.
\end{aligned}$$

where we considered $A = \delta + v + r$ and $A_T = \delta + v_T + r_T$. The rate A is the inverse of the mean time spent in the infected compartment when no treatment is applied, and A_T is the inverse of the mean time spent in the treated infected compartment. To determine the conditions for the stability of the DFE, we determine the eigenvalues of the Jacobian at the DFE, which is given by

$$J = \begin{bmatrix} -\delta & -\frac{\Lambda\beta}{\delta} & -\frac{\Lambda\beta(1-c_T)}{\delta} & 0 \\ 0 & \frac{\Lambda\beta}{\delta} - A - \theta & \frac{\Lambda\beta(1-c_T)}{\delta} & 0 \\ 0 & \theta & -A_T & 0 \\ 0 & r & r_T & -\delta \end{bmatrix}. \tag{2}$$

The characteristic polynomial of the Jacobian is

$$P_J(X) = (\delta + X)^2 \left(X^2 + X(A + \theta - \frac{\Lambda\beta}{\delta} + A_T) - (A_T(\frac{\Lambda\beta}{\delta} - A - \theta) + \theta\frac{\Lambda\beta(1-c_T)}{\delta}) \right). \tag{3}$$

The DFE is stable if all the eigenvalues are negative, which is equivalent to the condition

$$\frac{(A - A_T + \theta - \frac{\Lambda\beta}{\delta})^2 + 4\theta\frac{\Lambda\beta(1-c_T)}{\delta}}{(A + A_T + \theta - \frac{\Lambda\beta}{\delta})^2} < 1. \tag{4}$$

This inequality can be rewritten as

$$\frac{\Lambda\beta}{\delta}\frac{A_T + (1-c_T)\theta}{A_T(A+\theta)} = \frac{S^{eq}(DFE)}{S^{eq}(EE)} < 1. \tag{5}$$

This ratio corresponds to the basic reproduction number, which is the number of secondary infections that arise after an infected individual is placed in an environment consisting only of susceptible hosts. The stable equilibrium can be obtained by comparing the values of number of susceptible individuals at the DFE and the EE : The DFE is stable if $S^{eq}(DFE) < S^{eq}(EE)$ and the EE is stable if $S^{eq}(EE) < S^{eq}(DFE)$.

Text B: Equilibrium analysis with pathogen resistance to treatment

We consider the model with emergence of resistance, where a cost of transmission is associated to resistance to treatment

$$\begin{aligned}
\dot{S} &= \Lambda - \delta S - \beta S(I + (1 - c_{ROL})T_{ROL} + (1 - c_R)I_R) \\
\dot{I} &= \beta S(I + (1 - c_{ROL})T_{ROL}) - (\delta + v)I - rI - \theta_{ROL}I \\
\dot{T}_{ROL} &= \theta_{ROL}I - (\delta + v_{ROL})T_{ROL} - r_{ROL}T_{ROL} - s_R I_{ROL} \qquad (6) \\
\dot{I}_R &= \beta(1 - c_R)SI_R + s_R T_{ROL} - (\delta + v_R)I_R - r_R I_R \\
\dot{R} &= rI + r_{ROL}T_{ROL} + r_R I_R - \delta R.
\end{aligned}$$

There are three equilibria for the model with pathogen resistance to treatment. Two equilibria are an extension of the previous case : the DFE

($S^{eq}(DFE) = \frac{\Lambda}{\delta}$ and no infected) and the EE

$$\begin{aligned}
S^{eq}(EE) &= \frac{A'_{ROL}(A+\theta_{ROL})}{\beta\left(A'_{ROL}+(1-c_{ROL})\theta_{ROL}\right)} \\
I^{eq}(EE) &= \delta\frac{S^{eq}(DFE)-S^{eq}(EE)}{A+\theta_{ROL}+s_R\frac{\beta(1-c_R)\theta_{ROL}}{A'_{ROL}(A_R-\beta(1-c_R)S^{eq}(EE))}S^{eq}(EE)} \\
T^{eq}_{ROL}(EE) &= \frac{\theta_{ROL}}{A'_{ROL}}I^{eq} \qquad (7)\\
I^{eq}_{R}(EE) &= \frac{s_R}{A_R-\beta(1-c_R)S^{eq}(EE)}\frac{\theta_{ROL}}{A'_{ROL}}I^{eq} \\
R^{eq}(EE) &= \frac{1}{\delta}\left(r+r_{ROL}\frac{\theta_{ROL}}{A'_{ROL}}+r_R\frac{s_R}{A_R-\beta(1-c_R)S^{eq}(EE)}\frac{\theta_{ROL}}{A'_{ROL}}\right)I^{eq}
\end{aligned}$$

where we use

$$A'_{ROL} = \delta+v_{ROL}+r_{ROL}+s_R = A_{ROL}+s_R \qquad (8)$$

and

$$A_R = \delta+v_R+r_R. \qquad (9)$$

Clearly, the existence of the EE requires that $S^{eq}(DFE)-S^{eq}(EE)\geq 0$ and $A_R-\beta(1-c_R)S^{eq}(EE)\geq 0$.

The third equilibrium corresponds to the case where all infected individuals are resistant to treatment (Resistant Equilibrium, RE). The equilibrium is given in this case by $I^{eq}(RE) = T^{eq}_{ROL}(RE) = 0$ and

$$\begin{aligned}
S^{eq}(RE) &= \frac{A_R}{\beta(1-c_R)} \\
I^{eq}_{R}(RE) &= \delta\frac{S^{eq}(DFE)-S^{eq}(RE)}{A_R} \qquad (10)\\
R^{eq}(RE) &= r_R\frac{S^{eq}(DFE)-S^{eq}(RE)}{A_R}.
\end{aligned}$$

Similarly to the EE, the existence of the RE requires that $S^{eq}(RE) < S^{eq}(DFE)$. A further study of the Jacobian matrices at the three equilibria gives a simple criteria to determine which equilibrium the system has reached. The

equilibrium can be determined by comparing the number of susceptible individuals at the three different equilibria. The system reaches the equilibrium for which the number of susceptible individuals is minimal. For instance the RE is stable if

$$S^{eq}(RE) < S^{eq}(DFE) \text{ and } S^{eq}(RE) < S^{eq}(EE). \tag{11}$$

Text C: Population-level impact of imperfect tolerance-based treatment.

We now explore more parameter sets, and notably, we study the cases where treatment is not perfect ($v_{TOL} > 0$). We show in this section that a perfect treatment is not always better than an imperfect treatment, and that this depends on the type of infection and on coverage. To explore the effect of using an imperfect treatment on the population, we focused on the effect of different values of v_{TOL} on the mortality.
In the case of an acute infection, mortality is always decreasing with coverage and the most powerful treatment should always be preferred (Fig C panel A). The case of chronic infection is much more complex (Fig C panel B). The case examined in the manuscript (Fig 1) corresponds to a perfect treatment $v_{TOL} = 0$. In this case, if it is possible to target a large fraction of the population, then this treatment could be advantageous, while a low coverage could increase mortality. In the case of imperfect treatment (for instance $v_{TOL} = 0.6v$), population mortality always increases with treatment.

Text D: Why are the population-level effects more pronounced for chronic than for acute infections?

The effects of TOL are more pronounced for chronic than for acute infections. To study why this is the case, we focused on incidence, which only slightly increases with the coverage in acute infections but increases a lot in chronic infections. We examine the ratio of the incidence when there is 100% coverage to the incidence in the absence of treatment. After some simple derivations we arrive at the result:

$$\frac{Incidence(\varphi = 1)}{Incidence(\varphi = 0)} = \frac{R_0 - \frac{\delta + v_T + r_T}{\delta + v + r}}{R_0 - 1}. \tag{12}$$

In the numerical applications, we considered mostly the case $R_0 = 2$. With $v_{TOL} = 0$ and $r_{TOL} = r$, we obtain that the incidence ratio is

$$\frac{Incidence(\varphi = 1)}{Incidence(\varphi = 0)} = 1 + \frac{v}{\delta + v + r}. \tag{13}$$

The acute infection is characterized by the death rate being much lower than the recovery rate ($v << r$). In chronic infections it is the opposite ($v >> r$). This illustrates that the epidemiological feedback, which is mainly due to infectious individuals staying in the population, is amplified in chronic infections where people recover very slowly or not at all. The effect of treatment on the population is less pronounced for acute even though the mortality rate is larger. It is because the key factor here is the ratio between the mortality and recovery rates rather than the absolute value of the mortality.

Text E: Comparison of prophylactic and post-exposure treatments

To compare prophylactic and post-exposure treatments, we set up an epidemiological model in which a fraction of individuals are treated when they enter the population by immigration or birth. The model is described by the following system of ordinary differential equations:

$$
\begin{aligned}
\dot{S}_P &= \varphi\Lambda - \beta S_P(I_P + I_U) - \delta S_P \\
\dot{S}_U &= (1-\varphi)\Lambda - \beta S_U(I_P + I_U) - \delta S_U \\
\dot{I}_P &= \beta S_P(I_P + I_U) - (\delta + v_P + r_P)I_P \\
\dot{I}_U &= \beta S_U(I_P + I_U) - (\delta + v + r)I_U \\
\dot{R} &= r_P I_P + r I_U - \delta R.
\end{aligned}
\tag{14}
$$

Here, S_P is the number of treated susceptible individuals, S_U the number of untreated susceptible individuals, I_P is the number of treated infected individuals, I_U the number of untreated infected individuals, and R the number of recovered individuals. The parameter φ describes the fraction of the individuals that is treated upon birth or migration into the population of hosts. To compare the effects of a tolerance-based treatment applied pre- or post-exposure, we varied the treatment coverage and measured the mortality, prevalence and incidence of the infections, by choosing the same parameters as for post-infection treatment of acute and chronic infections. We obtain exactly the same results for pre- and post-exposure treatments at equilibrium, but these results are achieved for different coverage values. Thus, to achieve the same outcome, a larger absolute number of individuals must be treated, because both infected and non infected individuals are indifferently concerned.

There are no epidemiological benefits to prophylactic treatment, but this difference in coverage brings logistic and pharmacological downsides. First, the number of people that need to be treated to obtain the same results are much lower for tolerance-based treatment than for prophylaxis. Moreover,

individuals will need to receive prophylaxis for a longer time than if they were to receive TOL during the epidemic. Thus, TOL is more cost-beneficial than prophylaxis according to our analysis.

Text F: Impact of the different treatments on the basic reproduction number

In this section, we derive the variation of the basic reproduction number R_0 for various treatments. The basic reproduction number is the number of secondary infected that arise after an infected is placed in an environment consisting only of susceptible individuals.

We now study the variation of $R_0(\theta)$ when the treatment rate θ varies. We compute the derivative of R_0 with regard to θ and obtain

$$R_0'(\theta) = \frac{\Lambda\beta((1-c_T)A - A_T)}{\delta_S A_T (A+\theta)^2}. \tag{15}$$

Whether the R_0 increases or decreases with the treatment depends on the sign of $(1-c_T)A - A_T$. In the case of ROL, we considered that $c_{ROL} = 1$, $v_{ROL} = v$ and $r_{ROL} > r$, which leads to $c_{ROL}A - A_{ROL} = r - r_{ROL} < 0$. Hence $R_0'(\theta) < 0$ for ROL. For TOL, $c_{TOL} = 1$, $v_{TOL} = 0$ and $r_{TOL} = r$, and thus $R_0'(\theta) > 0$. Because for the DFE to be stable it is equivalent for the R_0 to be smaller than 1, we conclude that increasing the treatment rate θ for ROL can lead to the DFE, while it is not possible for TOL. If there is a cost of transmission TOL can lead to a lower incidence of the infection. The value of c_{TOL} such that $R_0' = 0$ is obtained for

$$(1-c_{TOL})A - A_{TOL} = (1-c_{TOL})(\delta + r + v) - \delta - r = 0, \tag{16}$$

which is attained for

$$c_{TOL} = \frac{v}{\delta + r + v}. \tag{17}$$

With the chosen parameters, the minimal cost that leads to a decrease of the R_0 is $c_{TOL} = 0.02$ for acute infections, $c_{TOL} = 0.875$ for chronic infections.

In the case of a treatment combination TOL+ROL, we have $r_{TOL+ROL} > r$, $v_{TOL+ROL} = 0$, and no cost of transmission $c_{TOL+ROL} = 0$. The derivative of R_0 has the same sign as $(1-c_T)A - A_T = v + r - r_{TOL+ROL}$. Depending on the relative values of the parameters, the sign of R_0' can thus be positive or negative. We can similarly evaluate the cost of transmission that would lead the R_0 to decrease with an increased treatment. We obtain that the threshold value of the cost of transmission is

$$c_{TOL+ROL} = \frac{v + r - r_{TOL+ROL}}{\delta + r + v}. \tag{18}$$

Because the recovery rate in the TOL+ROL treatment is larger than in the absence of treatment, $v+r-r_{TOL+ROL} < v$. The cost of transmission required for a decrease of the R_0 is lower for the TOL+ROL than for the TOL.

Interestingly, these results on the cost of transmission required to reduce incidence are independent on the value of β.

Text G: Can we curb disease incidence with transmission control?

In this section we extend the results of supplementary Text F, by examining the effect of changing the transmission rate of treated individuals. We plotted for both acute and chronic infections, the incidence, prevalence and disease-mortality when the cost of transmission c_{TOL} varies (see eq. 1 in Text A of the supporting information). We obtain that for acute infection, controlling transmission significantly decreases prevalence and incidence and to a lesser extent mortality. For chronic infections, the impact of transmission control is very limited for all three criteria (Fig. D).

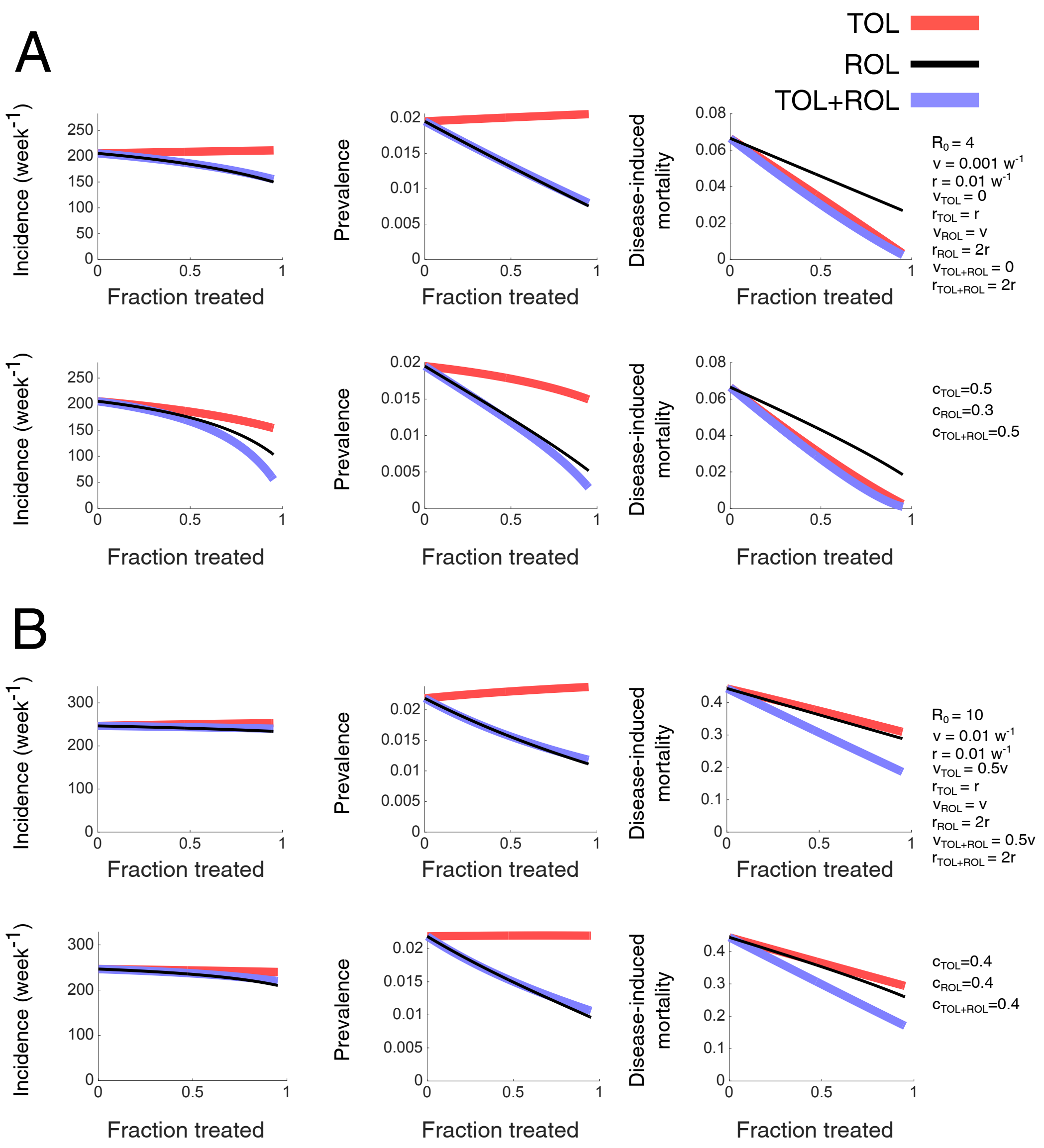

Figure A: **Robustness of the results to parameter changes.** Equilibrium values of the incidence, prevalence and mortality for two different infections (A) and (B). The death rate without a disease is set to $\delta = 1/70$ years^{-1} and Λ is determined so that the ratio Λ/δ is equal to a fixed population size in the case of no epidemic. We choose $\Lambda = 10^6\delta = 1.43\ 10^4$ years^{-1}. In the top row of each panel, the costs of transmission c_{TOL}, c_{ROL} and $c_{TOL+ROL}$ are equal to 0, and are changed for comparison in the bottom rows.

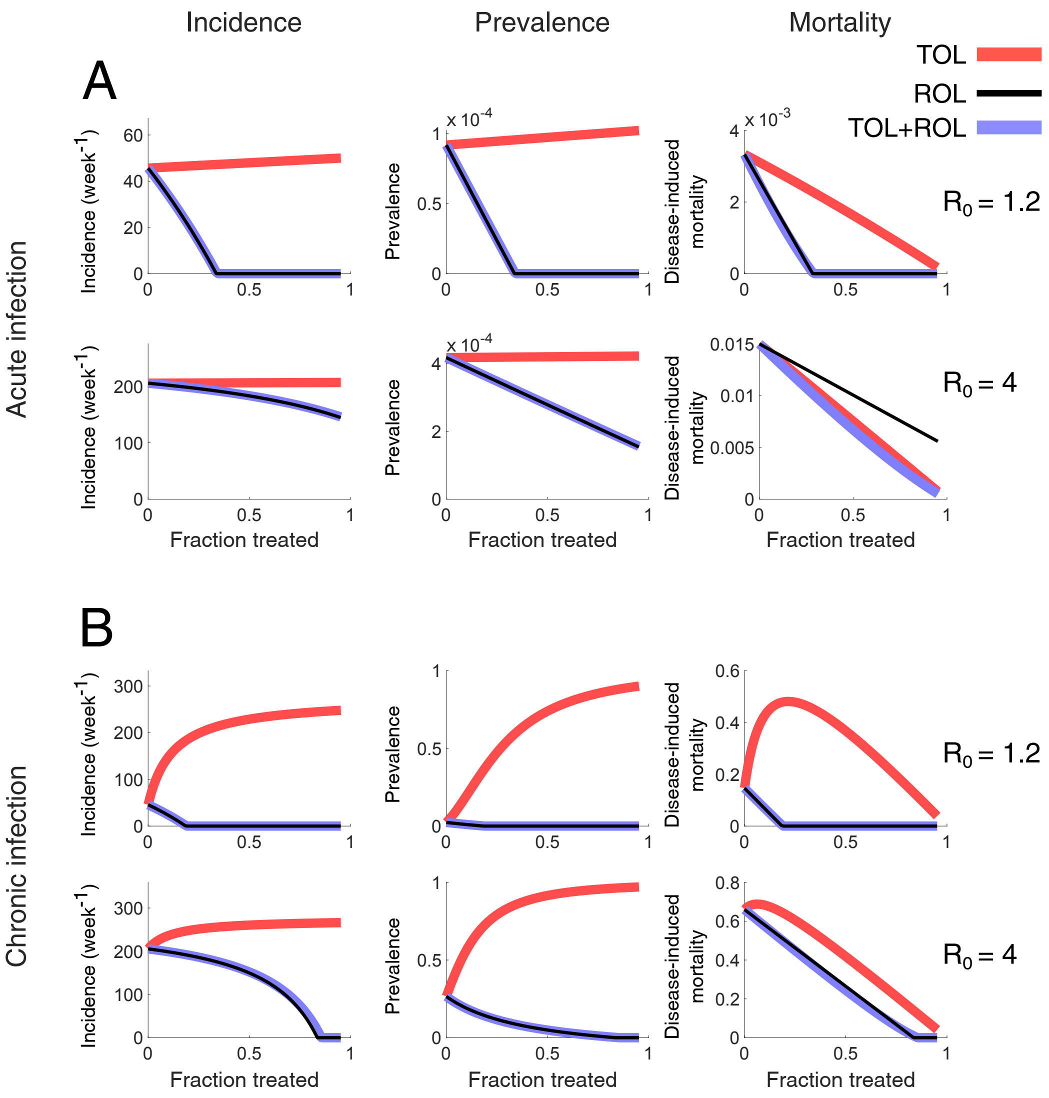

Figure B: **Effect of R_0 on the severity of infection.** Equilibrium values of the incidence, prevalence and mortality for acute (A) and chronic (B) infections. Parameters are given in the Methods section. The reproduction number R_0 is changed compared to Fig. 1, and is here $R_0 = 1.2$ (top rows) and $R_0 = 4$ (bottom rows).

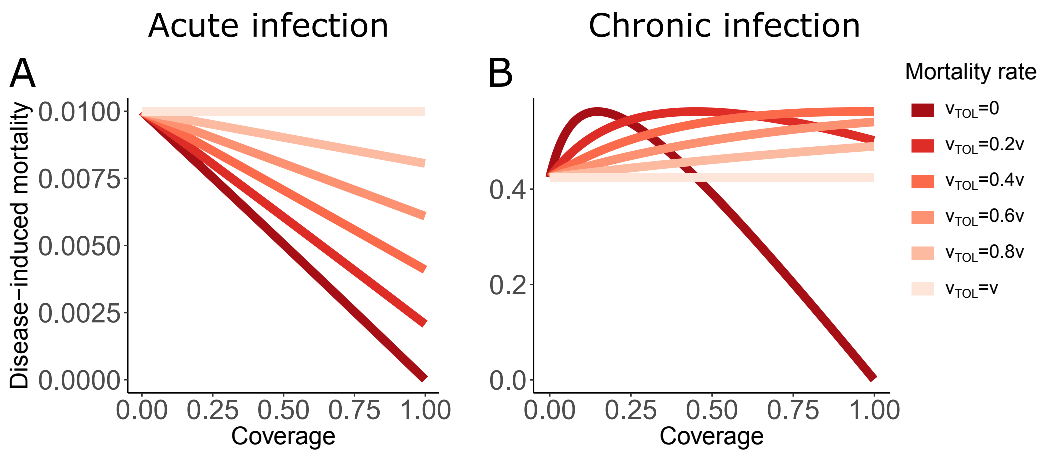

Figure C: Effect of the relative values of v_{TOL} and v on the disease-induced mortality, for acute (A) and chronic (B) infections. Colors indicate the value of the ratio v_{TOL}/v from perfect protection by TOL ($v_{TOL} = 0$, dark red) to no advantage of using TOL ($v_{TOL} = v$, light red).

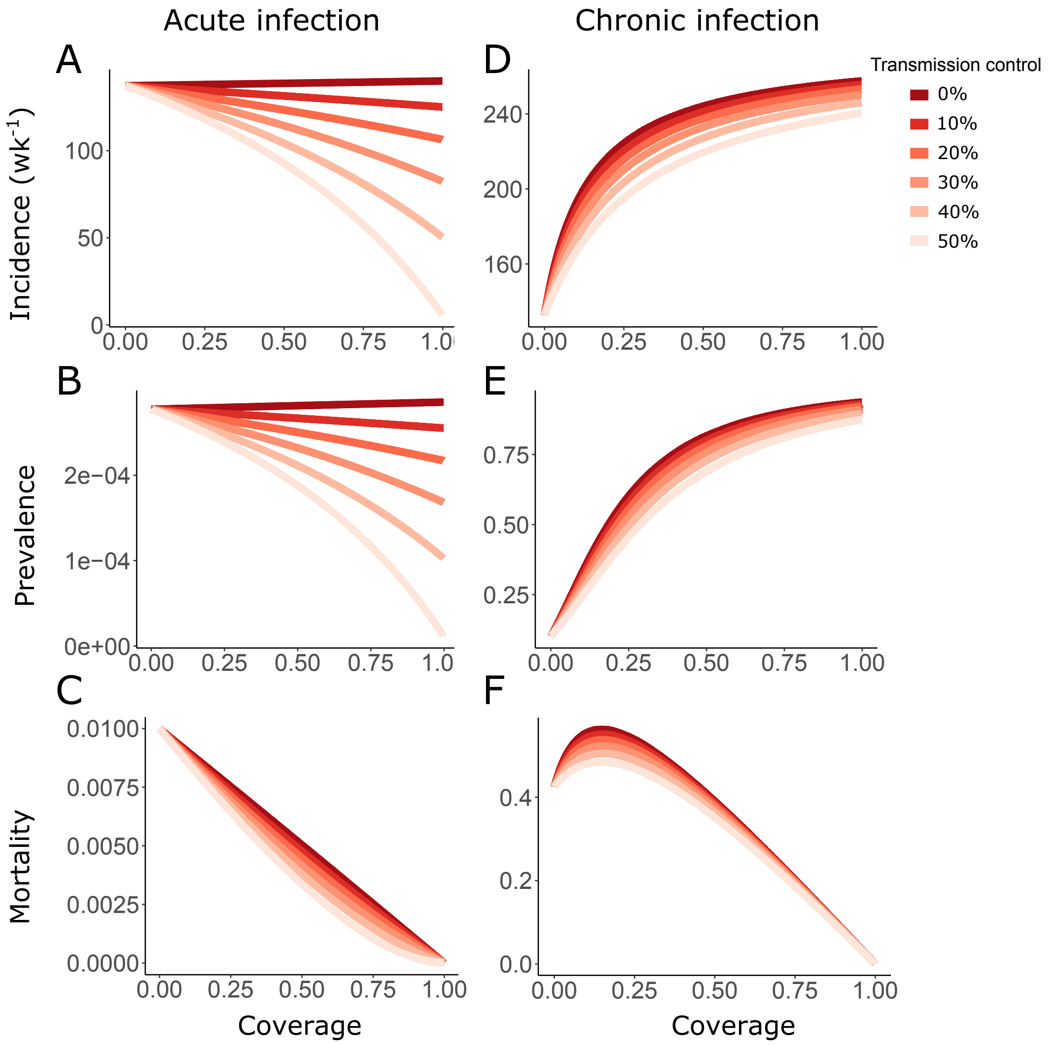

Figure D: Effect of transmission control on prevalence, incidence and mortality. A transmission cost modifies the transmission coefficient of treated individuals, for acute (A-C) and chronic (D-F) infections. Colors indicate the reduction in the transmission coefficient from β to β_{TOL}, from no control ($\beta_{TOL} = \beta$, dark red) to a 50% reduction in transmission (light red).
